# Pharmacological Neuroprotection after Perinatal Hypoxic-Ischemic Brain Injury

**DOI:** 10.2174/157015910793358150

**Published:** 2010-12

**Authors:** Xiyong Fan, Annemieke Kavelaars, Cobi J Heijnen, Floris Groenendaal, Frank van Bel

**Affiliations:** 1 Department of Neonatology, University Medical Center, Utrecht, the Netherlands; 2 Laboratory for Neuroimmunology and Developmental Origins of Disease (NIDOD), University Medical Center, Utrecht, the Netherlands; 3 Department of Paediatrics, Peking University First Hospital, Beijing, China

**Keywords:** Brain, hypoxia, ischemia, neonate, neuroprotection, pharmacology.

## Abstract

Perinatal hypoxia-ischemia (HI) is an important cause of neonatal brain injury. Recent progress in the search for neuroprotective compounds has provided us with several promising drugs to reduce perinatal HI-induced brain injury. In the early stage (first 6 hours after birth) therapies are concentrated on prevention of the production of reactive oxygen species or free radicals (xanthine-oxidase-, nitric oxide synthase-, and prostaglandin inhibition), anti-inflammatory effects (erythropoietin, melatonin, Xenon) and anti-apoptotic interventions (nuclear factor kappa B- and c-jun N-terminal kinase inhibition); in a later stage stimulation of neurotrophic properties in the neonatal brain (erythropoietin, growth factors) can be targeted to promote neuronal and oligodendrocyte regeneration. Combination of pharmacological means of treatment with moderate hypothermia, which is accepted now as a meaningful therapy, is probably the next step in clinical treatment to fight post-asphyxial brain damage. Further studies should be directed at a more rational use of therapies by determining the optimal time and dose to inhibit the different potentially destructive molecular pathways or to enhance endogenous repair while at the same time avoiding adverse effects of the drugs used.

## INTRODUCTION

Despite important progress in obstetric and neonatal care during the last decades, perinatal hypoxia-ischemia (HI) or birth asphyxia is still one of the most important causes of neonatal brain injury and the associated adverse developmental outcome [1, 2]. Currently, treatment options for post-asphyxial reperfusion/reoxygenation injury of the brain are largely supportive with prompt recognition and treatment of seizures, normalization of blood glucose levels, optimizing blood gases and blood pressure [3]. Recently, experimental and clinical studies have shown that moderate hypothermia with a reduction of body temperature to 33-to-34 ^°^C, if started within the first 6 h after birth, provides moderate neuroprotection [4-7]. This therapy has now been accepted in clinical practice as a strategy to fight post-asphyxial brain damage [8]. It is conceivable that outcome can further improve when hypothermia is combined with other (pharmacological) means of neuroprotection or with stimulation of regenerative processes in the neonatal brain. Damage due to perinatal HI in the premature infant is different from that in the full term newborn. In the premature infant, HI brain injury mainly involves pre-myelinating oligodendrocytes and white matter injury. However, this review will not discuss post-HI brain damage to the premature infant.

We will first summarize and briefly discuss the potentially destructive molecular pathways set in motion upon reperfusion and reoxygenation of the brain of the asphyxiated full-term neonate [9]. Subsequently, we will describe which pharmacological interventions can be considered to inhibit these destructive pathways including the most optimal postnatal moment to do so. 

## MOLECULAR MECHANISMS OF REPERFUSION INJURY TO THE BRAIN AFTER PERINATAL HI

Post-asphyxial reperfusion injury to the brain is caused by a cascade of molecular reactions as summarized below in order of their occurrence:

### 1. Calcium Influx and Free Radical Formation

During HI, ATP formation is impaired due to hypoxia and immediately after reperfusion and reoxygenation reactive oxygen species are produced. Together, these processes cause an excessive influx of calcium stimulated by opening of voltage-regulated calcium channels which lead to release of neurotransmitters such as glutamate. Glutamate can activate receptor-regulated (N-methyl-D-aspartate [NMDA]) calcium channels thereby further increasing calcium. This triggers enhanced production of free radicals and activation of lipases, proteases, and endonucleases. As a consequence, releasing of free fatty acids, especially arachidonic acid, will activate cyclooxygenase and will catalyse the formation of prostaglandins which will liberate among other things superoxide free radicals. In addition, formation of oxygen free radicals is also enhanced *via *metabolizing hypoxanthine formed during the actual period of HI to uric acid. Collectively, these processes will lead to a surge of the superoxide free radical, which plays a central role in further production of free radicals and other toxic compounds, see also below [10-12].

### 2. Free Iron Accumulation and Fenton Reaction

During the actual period of HI, protein-bound iron within the neuronal and microglial cells will be liberated from its binding proteins, especially because of the low intracellular pH.Non-protein bound iron (NPBI) or free iron will accumulate, which is an important pro-radical. Upon reperfusion and reoxygenation, NPBI will react with superoxide-derived hydrogen peroxide (see above) to form the very toxic hydroxyl free radical, the so-called Fenton reaction [13]. NPBI has been related to excessive brain damage in the immediate post-HI period in both experimental [14] and clinical [15] studies.

### 3. Nitric Oxide Synthases and Brain Injury

Nitric oxide (NO) is a free radical produced by nitric oxide synthases (NOS) that are expressed in the brain in neurons, astrocytes, and endothelial cells and can be induced in microglia. There are three isoforms of NOS [16]: endothelial or eNOS, neuronal or nNOS, which are constitutionally expressed forms of NOS that produce NO in moderate amounts in response to increased intracellular calcium. An inducible form or iNOS can also be expressed in the brain. eNOS is thought to have a neuroprotective function *via *enhancing perfusion of the brain if necessary [17]. Upon reperfusion and reoxygenation after perinatal HI, however, eNOS and nNOS are activated leading to excess production of NO. Moreover, in a later stage (see below), continuous production of NO occurs *via *upregulation of iNOS in infiltrating neutrophils, macrophages and microglia. NO then can react with superoxide (see above) to form the toxic peroxynitrite which can contribute to further damage to brain tissue [18-21]. 

### 4. Pro- and Anti-Inflammatory Activity

Three-to-12 hours after reperfusion and reoxygenation an inflammatory response, probably induced by excessive free radical production and high levels of extracellular glutamate, will be activated and pro- and anti-inflammatory cytokines such as TNF-α, IL-1, IL-6, IL-8 and IL-10 are produced [22]. The activation of two transcription factors, *i.e.* Nuclear Factor kappa B (NFκB) and c-Jun N-terminal kinase (JNK) play a central role in the post-HI inflammatory process. In addition, these transcription factors can regulate expression of pro- and anti-apoptotic proteins and thus can contribute to damage (see below) or neuroprotection [23-25]. 

### 5. Apoptosis Activation

As indicated above, apoptotic activity contributes to brain damage in the neonate and is an important pathway in the process of delayed neuronal death [26]. Apoptosis is an energy-dependent process and ATP is required for apoptosome formation and subsequent caspase activation [27]. Caspases and especially the executioner caspase-3 are activated in this process and bring about most of the changes that characterize apoptotic cell death [28]. Activated caspase-3 is expressed at higher levels in the developing brain after perinatal HI, giving rise to the assumption that apoptotic mechanisms of neuronal cell death seem to be more important in neonatal brain injury compared to adults [29].

### 6. Downregulation of Growth Factors

It has been proposed that inflammatory activity in the brain, together with the excessive production of toxic compounds like peroxynitrite and free radicals induce downregulation of the formation of neurotrophic factors and neurogenesis. Since the ability of the developing brain to recover after perinatal HI may well depend on production of neurotrophic and neurite-outgrowth promoting factors, downregulation of these growth factors may play an important role in delayed brain damage (up to several weeks) after perinatal HI [30]. 

Fig. (**1**) shows proposed injuring mechanisms induced upon and after reperfusion / reoxygenation after perinatal hypoxia-ischemia.

## PHARMACOLOGICAL NEUROPROTECTIVE STRATEGIES

Based on the mechanisms of perinatal HI brain injury, current therapeutic studies mainly focus on the pharmacologic targets we mentioned above. Since moderate hypothermia is developing into a clinical therapy, studies to investigate the effect of pharmacological interventions in combination with hypothermia are now also initiated. These pharmacologic therapies can start at different points of time after the actual HI insults with or without hypothermia depending on to the mechanism of action (neuroprotection versus repair mechanisms). In addition, treatment with stem cells to enhance repair is a promising intervention [31, 32] to repair HI brain damage.

### 1. Early Post-HI Period (up to 6 Hours after Reperfusion/Reoxygenation)

The early post-HI period contains an important therapeutic window [33] and the main pharmacological interventions are dependent on anti-oxidative, anti-inflammatory, and/or anti-apoptotic properties.

#### Voltage-Regulated and Receptor-Regulated Ion Channel Blockers

Reduction of calcium influx into neuronal cells by blockage of voltage-regulated ion channels has extensively been investigated. Calcium blockers including nicardipine and flunarizine gave encouraging results in experimental studies [34, 35]. However, a clinical study with nicardipine in term neonates suffering from perinatal asphyxia had to be discontinued because of severe hypotension [36]. Blocking NMDA-receptors (*i.e.* glutamate regulated ion channels) with magnesium was investigated in clinical trials, but without beneficial effects and severe hemodynamic adverse effects were sometimes reported [37-39]. A recently advocated approach is the use of the gaseous anesthetic Xenon, which is thought to act as an NMDA-receptor antagonist. Experimental studies in newborn animals show efficient neuroprotection, especially when administered in combination with moderate hypothermia [40, 41]. A major disadvantage of this intervention is that Xenon is very expensive and administration is rather complicated, requiring intubation and ventilation of the patient, and a high percentage of Xenon thereby reducing the maximum FiO_2_. 

#### Anti-Oxidative Therapies

The production of superoxide, the superoxide-derived hydrogen peroxide and hydroxyl radicals are mainly related to xanthine-oxidase, prostaglandin and NPBI production [42-44]. Allopurinol is a xanthine-oxidase inhibitor that at higher dosages is thought to chelate free iron molecules and to be a direct scavenger of hydroxyl radical [45]. This should be also true for oxypurinol, the even more efficient active metabolite of allopurinol [46]. Allopurinol was first recognized to have neuroprotective properties by Palmer *et al*. in a neonatal rat model of HI brain injury [47]. The data of this study were promising, although another study was less positive on the neuroprotective potential of allopurinol [33]. Human studies in asphyxiated term newborns are not very convincing concerning the neuroprotective effects of allopurinol and oxypurinol, showing at best a moderate reduction of free radical production. One study reported virtually no positive effect on neurodevelopmental outcome [48], whereas one study in asphyxiated term newborns reported an improvement of neurodevelopmental outcome after allopurinol treatment [49]. Most probably, in most clinical studies therapy is started too late to expect a reduction of xanthine-oxidase production [48]. A pilot study of our group on maternal allopurinol treatment in those mothers who were on the brink of delivery with fetal hypoxia, showed a reducing effect on biomarkers of neuronal damage and NPBI after allopurinol administration to the mother [50]. We are currently examining the effect of maternal allopurinol treatment on neonatal outcome in a similar set-up.

With respect to the pro-free radical properties of NPBI, several experimental studies have been performed in newborn animals of various species including pigs and rats with positive results [14, 51]. Up to now, however, free ion chelators, have never been tested in newborn babies to treat reperfusion/reoxygenation injury after perinatal HI.

Due to the toxic effects of excessive formation of NO free radical in the early reperfusion/reoxygenation phase, inhibition of NOS production may ameliorate perinatal brain damage after HI. Non-selective NOS inhibitors such as nitro-L-arginine administered during the early post-HI period have been reported to reduce free radical-mediated reperfusion injury to the neonatal brain [52, 53]. However, an increasing number of studies showed that non-selective NOS inhibitors, Due to the toxic effects of excessive formation of NO free radical in the early reperfusion/reoxygenation phase, inhibition of NOS production may ameliorate perinatal brain damage after HI. Non-selective NOS inhibitors such as nitro-L-arginine administered during the early post-HI period have been reported to reduce free radical-mediated reperfusion injury to the neonatal brain [52, 53]. However, an increasing number of studies showed that non-selective NOS inhibitors, especially those with prominent inhibitory effects on eNOS, prevent adequate post-HI brain perfusion, eventually leading to increased production of free radicals and thus aggravating brain damage [54-56]. Selective inhibition of nNOS and iNOS with the nNOS inhibitor 7-nitroindazole and the iNOS inhibitor aminoguanidine proved to be more promising as a neuroprtective strategy as has been shown in several studies in neonatal rats [57-59]. The compound 2-iminobiotin (2-IB), which has inhibitory effects on nNOS and iNOS *in vitro*, did have strong neuroprotective effects in a neonatal rat model of neonatal HI brain damage. Notably, however, only female and not male animals were protected against post-HI reperfusion damage to the brain [60, 61]. Moreover, the existing evidence suggests that the *in vivo* neuroprotective effect of 2-iminobiotin was not dependent on nNOS/iNOS inhibition [61, 62]. The exact mechanism of action of 2-IB remains to be determined, but it is clear that in females neuroprotection by 2-IB is associated with reduced activation of apoptotic pathways.

Finally, prostaglandin inhibition has been another important target to fight post-HI brain damage in the newborn. Indomethacin, a cyclooxygenase inhibitor, has been shown to reduce neonatal brain damage after perinatal HI in experimental studies [63]. Although indomethacin is currently used in preterm babies to reduce or prevent the occurrence of periventricular/intraventricular hemorrhages [64, 65] and can reduce white matter injury in these tiny infants [66, 67], it has not been used yet in the term infant to reduce reperfusion/reoxygenation injury of the brain after perinatal HI. 

#### Anti-Inflammatory Therapies

As mentioned above, the inflammatory pathway is activated after perinatal HI and therefore anti-inflammatory strategies are another meaningful tool to fight reperfusion injury to the newborn brain. 

Erythropoietin (EPO), which was first recognized as a humoral mediator involved in the maturation and proliferation of erythroid progenitor cells [68], has recently been recognized as a neuroprotective agent in the brain of a variety of mammals including humans [68-72]. Mostly recombinant Human EPO (rhEPO) has been used in these studies. One of the possible mechanisms of its neuroprotective effects is an anti-inflammatory effect after binding to its receptor (EPOR) which is expressed on several types of brain cells including astrocytes and microglial cells [73, 74]. Intraperitoneal and subcutaneous administration of EPO in newborn animals after HI improved neurobehavioral outcome [75, 76] and administration to humans also had neuroprotective effects [72]. Also negative results are reported in both clinical and experimental studies [77, 78]. Gender effects are also reported to contribute to the neuroprotective role of EPO. In a rat model of neonatal stroke, EPO reduced the infarct volume and improved long-term behavioral outcome more significantly in females than in males [79]. An ongoing study in mice pups of our own group also suggests similar gender dependent neuroprotection in females after neonatal HI (unpublished data, Fan *et al*.). 

Melatonin, the major secretory product of the pineal gland, mainly mediates circadian rhythmicity and seasonality [80]. Recent studies reported that melatonin has a neuroprotective effect during perinatal HI-induced brain injury. Using a P7 rat HI model, it was shown that sensorimotor asymmetry and learning deficits were significantly reduced after administration of melatonin before or up to 10 min after HI [81]. Histologically, brain injury was significantly attenuated in the melatonin-treated group. In a fetal sheep model with umbilical cord occlusion, melatonin had anti-inflammatory effects as it reduced microglial cell activation [82]. The anti-inflammatory effect of melatonin may be mediated by preventing the translocation of NF-κB to the nucleus, thus reducing the upregulation of pro-inflammatory cytokines [83]. To date no human studies are known investigating its neuroprotective effects after perinatal HI.

Moreover, our own recent studies have shown that treatment of neonatal rats after HI with etanercept, a soluble TNF-α receptor functioning as a TNF-α inhibitor, also has neuroprotective effects [84]. 

#### Anti-Apoptotic Strategies

As we mentioned above, NFκB is a ubiquitously expressed transcription factor that regulates expression of inflammatory genes and of genes involved in apoptosis. TAT-coupled (a method for transducing proteins into cells) NFκB essential modulator Binding Domain (NBD)-peptide, a specific NFκB inhibitor, was reported to rapidly distribute to the brain and inhibit cerebral NFκB activation when administered intraperitoneally after neonatal HI in p7 rats. TAT-NBD treatment prevented upregulation of p53 and activation of caspase-3 after HI [23]. TAT-NBD treatment strongly reduced histological damage when administered within 6 h after HI. Our most recent data demonstrate that at this histological improvement was associated with restoration of sensorimotor and cognitive abnormalities. Surprisingly, NFκB treatment did not reduce cerebral cytokine production despite the marked protective effects. Activation of the c-Jun N-terminal kinase (JNK) pathway is also involved in neonatal brain injury [25]. The specific JNK inhibitor TAT-JBD, which is the TAT coupled JNK binding domain of JNK-interacting protein-1 (also known as L-JNK-I) also has neuroprotective effects. Recent studies reported that JBD improved both short term and long term histological and behavioral outcomes in neonatal rats after HI [84, 85]. Similar to what was observed for the NFκB inhibitor, the protective effect of JBD occurred independently of inhibition of cytokine production [85]. Notably, prolonged inhibition of the JNK pathway as induced by administration of the D-isomer of JNK-I or of the NFκB pathway by repeated administration of NBD at 0, 6 and 12h after HI did not improve outcome or even had some adverse effects [24, 86, 87]. 

#### Hyperbaric Oxygen Therapy

The earliest insight into the therapeutic effect of hyperbaric oxygen (HBO) originated from the observation that HBO was capable of decreasing edema and necrosis in ischemic skeletal muscle [88]. Although the optimal application and therapeutic effect of HBO therapy in neonatal HI brain injury remains controversial [89], studies on the effect of HBO are still being carried out [90]. In a P7 rat model, administration of HBO for 1h after HI significantly improved sensorimotor function during behavioral test and reduced the loss of brain volume [91]. The detailed mechanisms of neuroprotection by HBO are not clear yet. Existing studies indicated that neuroprotection was associated with reducing polymorphonuclear leukocytes (PMNL) adhesion [92], downregulating endothelial cell adhesion molecules (CAM) expression [93], inhibiting iNOS production while inducing eNOS production [93, 94], upregulating antioxidant enzyme activity [95] and improving cellular energetics [96]. Furthermore, HBO is capable of suppressing mitochondrial apoptotic pathways via reducing cytoplasm cytochrome c levels, decreasing caspase enzyme activity and upregulating the ratio of Bcl-2 (an anti-apoptotic gene) and Bax (a pro-apoptotic gene) expression [97]. HBO also contributes to brain cell proliferation [98]. In a P7 rat hypoxic-ischemic model, the treatment of HBO could be delayed until 12 h after HI, while the effect decreased 24 h after HI [99].

### 2. Treatment Modalities Later in the Post-HI Period

The intrinsic ability of the immature brain to reduce (post) HI-induced damage is also dependent on production of trophic factors and endogenous regenerative activity. In view of the capacity of the neonatal brain to repair damage, stimulation of endogenous repair processes has also been suggested as a possible intervention in neonatal HI-brain damage.

#### Trophic Factor Therapy

Several trophic or growth factors have been examined in the context of neonatal HI brain injury: epidermal growth factor (EGF), insuline-like growth factor 1 (IGF-1) and brain-derived neurotrophic factor (BDNF) among other factors are very important for the appropriate development of the developing brain [100]. Downregulation of the expression (neurotrophic) growth factors is thought to play a pivotal role in (delayed) damage to the brain of the neonate after perinatal HI. Suppletion of deficient growth factors may therefore reduce or prevent delayed HI-induced brain damage. Trophic factors also stimulate neurogenesis by activation of the endogenous neural stem cells (NSC) residing in the subventricular zones and subgranular zone of the dentate gyrus, as shown in studies in rodent pups models [101, 102].

EPO has, besides its anti-oxidative and anti-inflammatory effects [74, 103], also quite strong neurotrophic abilities as was shown in experimental animal and *in vitro* studies: a 17-mer peptide sequence called epopeptide AB has been identified in the structure of EPO, and this peptide is considered to contain the neurotrophic properties [104]. *In vitro* this peptide can induce neural progenitor cell proliferation and prevents neuronal cell death [105]. In an adult rat stoke model, administration of EPO promoted neurogenesis in the subventricular zone *via *upregulation of BDNF expression [106]. By using embryonic a neural stem cell culture, studies showed that EPO enhanced formation of neurons [107]. In a P10 rat stroke model, EPO increased the percentage of newly generated neurons as well [108].

Other neurotrophic factors have also been investigated. IGF-1, an anabolic pleiotrophic factor, is essential for postnatal brain development [109]. It has been shown that IGF-1 is induced in neonatal brain after HI [110]. In a P7 rat model, intranasal administration of IGF-1 improved neurobehavioral performance, inhibited apoptotic cell death, and enhanced proliferation of neuronal and oligodendroglial progenitor cells after HI [111]. Basic fibroblast growth factor (bFGF), a polypeptide growth factor, has been shown to prevent NMDA-induced neurotoxicity in neonatal rats after HI [112]. Continuous intracerebroventricular injection with BDNF resulted in an increased number of surviving neurons in P8 rat brains after HI [113]. An important role with respect to neurotrophic reduction and repair of brain damage has been supposed for hypoxia-induced-factor 1 (HIF-1), which will be activated during hypoxia-ischemia and gives rise to the production of a series of transcriptional targets, of which EPO and vascular endothelial growth factor (VEGF) are important ones with respect to brain repair [114-116].

Neuronal stem cell transplantation is a potentially important therapy to reduce long-term brain damage after perinatal HI and have shown to improve motor- and behavioral outcome in adolescent and adult periods [31, 117, 118]. It appears from these studies that NSC’s can differentiate into neurons and oligodendrocytes and stimulate angiogenesis [31, 119].

Recent evidence suggests that stroke or HI-induced global brain damage can also be treated with stem cells from other sources than brain, such as mesenchymal stem cells (MSCs) [120, 121]. MSCs can easily be obtained from the bone marrow of healthy adults, placental tissue, umbilical cord stroma (Wharton's Jelly) and even from cord blood. Since MSCs are hardly immunogenic, these cells can also be used for allogeneic transplantation. After administration in the brain, these MSCs can express neuronal markers like NeuN and MAP-2, the astroglial marker GFAP, the microglial marker IB4 [122, 123]. Furthermore, It is known that MSC secrete several trophic factors that are known to contribute to neuroprotection including, colony-stimulating factor-1, stem cell factor, VEGF, basic fibroblast growth factor (bFGF), nerve growth factor (NGF) and brain-derived neurotrophic factor (BDNF) [124, 125]. Notably, recent findings indicate that intracranial administration of MSC as late as 3-10 days after the HI insult reduces histological damage and improves sensorimotor outcome in both neonatal mouse and rat HI model [31, 117, 118]. We also showed that MSC treatment stimulated formation of new neurons and oligodendrocyte and that newly formed cells were not of transplant origin. These findings indicate that MSC treatment enhances endogenous repair mechanisms in the brain [32].

### 3. Combination Therapy: More Effective than Single Therapy?

Although studies in rodents have identified many different molecular pathways as potential targets for neuroprotective strategies after perinatal HI, intervening in one particular pathway may not be sufficient to completely prevent brain injury because of the complex mechanisms of hypoxia-ischemia. Rather than a single therapy directed at one of the potentially destructive pathways, combinations of therapies intervening at different levels in the cascade might lead to more prominent reduction of brain injury. 

There is already some evidence that pharmacolgoical interventions combined with hypothermia have a stronger neuroprotective effect after HI than either one alone [41, 126]. Since it is conceivable that hypothermia postpones secondary energy failure, application of hypothermia immediately after the hypoxic event could prolong the window for pharmacotherapeutic intervention [6, 127]. In a P7 rat HI model, the hypothermia/Xenon combination conferred more protection as determined in behavioral tests and at the level of histology than either treatment alone [41]. In addition, protection was observed even when Xenon administration was delayed or applied for shorter period of time [128]. The hypothermia/EPO combination is used in out-of-hospital cardiac arrest and this combination can improve the survival rate in adults, but to date there are no data on combined EPO/hypothermia treatment in neonatal HI [129]. Administration of melatonin to adult rats during hypothermia promotes tissue oxygenation and enhances the body's resistance to hypothermia [130]. However, this combination has only been tested in adult studies, and there is no experience in neonatal models. An extremely important issue here is that each pharmacologic compound directed at a specific molecular pathway has its own optimal dose and optimal point in post-ictal time to achieve its optimal neuroprotective effect. It will be a major challenge to define the optimal time of application for each individual intervention and to design the best schedule to let them act in concert. 

Fig. (**2**) shows the possibilities for combination therapy and the most optimal point of time to start a specific treatment modality.

Fig. (**3**) shows the most important pharmacological agents investigated in experimental and clinical studies for treatment of reperfusion/reoxygenation injury to the neonatal brain after perinatal asphyxia.

## CONCLUSIONS AND RECOMMENDATIONS

In conclusion, rapid progress in neuroprotective pharmacology has already provided us with a wide selection of pathways that could be targeted for treatment of perinatal HI-induced brain injury. Combination of therapies may lead to a larger neuroprotective effect on the brain than single compound treatment and this possibility should be pursued further. 

Further research of promising pharmacologic interventions should be intensively performed and major attention should be given to reducing side effects and toxicity so that more and more therapies can be carried from animal experiments to clinical trials. Recent findings indicating that gender differences in sensitivities to both HI injury and drug treatment should be taken into account [131] and gender-specific therapies may provide more promising interventions for perinatal HI-induced brain injury. Finally, recent findings indicating that stimulation of endogenous repair mechanisms can have potent effects on both histological and behavioral are promising. These findings are especially important since repair mechanisms could be effectively activated even days to weeks after the insult.

## Figures and Tables

**Fig. (1):**
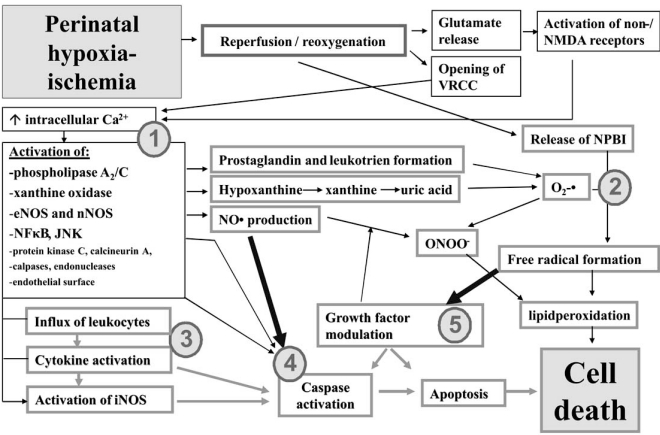
Possible injuring mechanisms induced upon and after reperfusion/ reoxygenation after perinatal hypoxia-ischemia. Reperfusion/ reoxygenation induces opening of ion channels which leads to influx of calcium into neurons and subsequent excessive neurotransmitters producing and activation of enzymatic reactions (1). The actions give rise to subsequent metabolisation of hypoxanthine, fatty acids originating from neuronal cell membranes, and of nitric oxide production and the Fenton reaction (2). This leads to excessive free radicals formation, lipidperoxidation and ultimate neuronal cell death. Somewhat later (6-12h after reperfusion/reoxygenation), the pro-inflammatory response occurs with activation of cytokines and iNOS production (3), leading to caspases activation (4), growth factor modulation (5) and apoptosis. *JNK=c-Jun N-terminal kinase; NMDA= N-methyl-D-aspartate; NO= nitric oxide; NOS= nitric oxide synthase; eNOS= endothelial NOS; nNOS= neuronal NOS; iNOS=inducible NOS, NPBI=non-protein bound iron; NFκB=Nuclear Factor kappa B; VRCC=voltage-regulated calcium channel.*

**Fig. (2):**
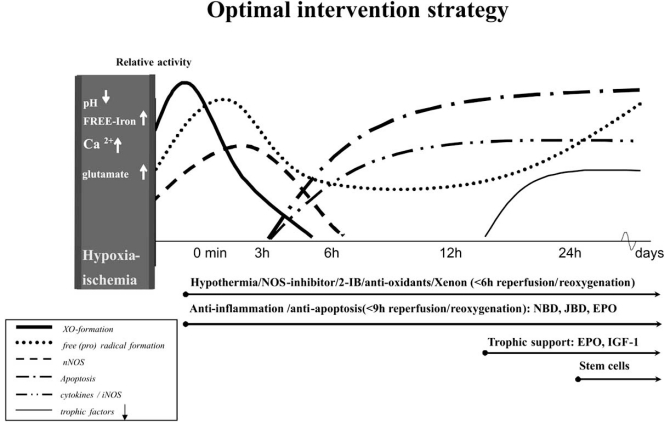
Possibilities for combination therapy and the potential optimal point of time to start a specific treatment modality. XO, free radicals and nNOS increase prenatally after hypoxia-ischemia and reach the peak immediately after reperfusion/reoxygenation. Then there is a quick decrease in these three kinds of factors during the first 6h after reperfusion/reoxygenation. However, there is a secondary increase of free radical formation 12h later. Cytokines, iNOS and apoptotic activity appear 3h after reperfusion/reoxygenation and gradually increase to the plateau at 6h. 12h after reperfusion/reoxygenation, the downregulation of trophic factors starts and is stabilized at 24h. Interventions of hypothermia/NOS-inhibitor/2-IB/anti-oxidants/Xenon should be started as early as possible, at least no later than 6h after reperfusion/reoxygenation. Interventions of anti-inflammation and anti-apoptosis should also start early and no later than 9h after reperfusion/reoxygenation. Trophic support with EPO and IGF-1 can be performed 12h after reperfusion/reoxygenation. Furthermore, stem cells are suggested to be carried out 24h later. *EPO=erythropoietin; IGF-1=insuline-like growth factor 1; 2-IB=2-iminobiotin; NOS= nitric oxide synthase; nNOS= neuronal NOS; TAT-JBD= TAT coupled JNK binding domain; TAT-NBD=TAT-coupled NFκB essential modulator binding domain; XO=xanthine-oxidase.*

**Fig. (3):**
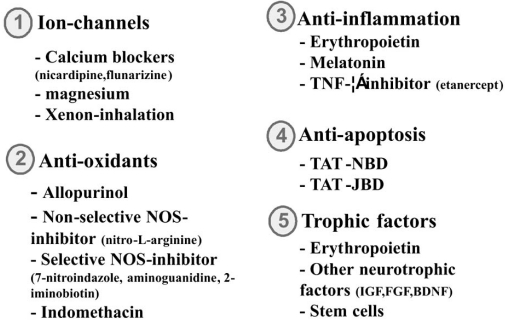
The most promising pharmacological interventions investigated in experimental and clinical studies on perinatal HI brain injury. *BDNF = brain-derived neurotrophic factor; FGF = Fibroblast growth factor; IGF = insuline-like growth factor; NOS= nitric oxide synthase; TAT-JBD= TAT coupled JNK binding domain; TAT-NBD=TAT-coupled NFκB essential modulator binding domain; TNF-α = tumor necrosis factor.*
